# CD44 enhances tumor aggressiveness by promoting tumor cell plasticity

**DOI:** 10.18632/oncotarget.3839

**Published:** 2015-04-15

**Authors:** Yvette W.J. Paulis, Elisabeth J.M. Huijbers, Daisy W.J. van der Schaft, Patricia M.M.B. Soetekouw, Patrick Pauwels, Vivianne C.G. Tjan-Heijnen, Arjan W. Griffioen

**Affiliations:** ^1^ Division of Medical Oncology, Department of Internal Medicine, GROW - School for Oncology and Developmental Biology, Maastricht University Medical Center, Maastricht, The Netherlands; ^2^ Department of Medical Oncology, Angiogenesis Laboratory, VU University Medical Center, Amsterdam, The Netherlands; ^3^ Laboratory of Pathology, University of Antwerp, Antwerp, Belgium; ^4^ Department of Biomedical Engineering, Soft Tissue Biomechanics and Engineering, Eindhoven University of Technology, Den Dolech, Eindhoven, The Netherlands

**Keywords:** cancer, CD44, ewing sarcoma, c-Met, vasculogenic mimicry

## Abstract

Aggressive tumor cells can obtain the ability to transdifferentiate into cells with endothelial features and thus form vasculogenic networks. This phenomenon, called vasculogenic mimicry (VM), is associated with increased tumor malignancy and poor clinical outcome. To identify novel key molecules implicated in the process of vasculogenic mimicry, microarray analysis was performed to compare gene expression profiles of aggressive (VM^+^) and non-aggressive (VM^−^) cells derived from Ewing sarcoma and breast carcinoma. We identified the CD44/c-Met signaling cascade as heavily relevant for vasculogenic mimicry. CD44 was at the center of this cascade, and highly overexpressed in aggressive tumors. Both CD44 standard isoform and its splice variant CD44v6 were linked to increased aggressiveness in VM. Since VM is most abundant in Ewing sarcoma tumors functional analyses were performed in EW7 cells. Overexpression of CD44 allowed enhanced adhesion to its extracellular matrix ligand hyaluronic acid. CD44 expression also facilitated the formation of vasculogenic structures *in vitro*, as CD44 knockdown experiments repressed migration and vascular network formation. From these results and the observation that CD44 expression is associated with vasculogenic structures and blood lakes in human Ewing sarcoma tissues, we conclude that CD44 increases aggressiveness in tumors through the process of vasculogenic mimicry.

## INTRODUCTION

Vasculogenic mimicry (VM) describes the process in which highly aggressive, plastic tumor cells gain characteristics normally restricted to endothelial cells. This transdifferentiation allows tumor cells to form matrix-rich vasculogenic tubular structures [1]. Through this means, tumor cells can contribute to blood circulation independent of angiogenesis. After the initial observation of VM in melanoma, evidence for its occurrence has been reported in other tumor types [2-7] and research has focused on identifying the molecular mechanism underlying this phenomenon [8]. Further understanding of this process specifically revealed that in e.g. glioblastoma it is the stem-like glioblastoma cell subset that is responsible for the capacity to transdifferentiate into endothelial-like cells. It was demonstrated that these cells gain the ability to activate transcriptional programs assumed to be restricted to endothelial cells [9, 10].

Ewing sarcoma is a rare but highly aggressive bone and soft tissue tumor that arises in children and young adults. In patients with localized disease, modern treatment modalities are able to achieve cure rates of approximately 70%. However, the prognosis of patients with metastatic Ewing sarcoma at time of diagnosis still remains inferior, indicating the limitations of current treatments strategies [11]. Previously, we demonstrated the presence of VM in Ewing sarcoma [7]. This phenomenon is associated with increased tumor malignancy and may contribute to tumor progression. CD44 is a transmembrane glycoprotein receptor originally identified to be involved in leukocyte adhesion and recirculation. At present, it is widely recognized as a pleiotropic molecule expressed by stem cells and involved in tumor metastasis [12, 13]. It is described to be a marker of epithelial-to-mesenchymal transition and also of tumor endothelium [14]. The main ligand for CD44 is hyaluronic acid. This extracellular matrix constituent functions as a microenvironmental cue to regulate cell behavior during processes like embryonic development, tissue remodeling, wound healing, inflammation, and tumor growth. In the latter process, hyaluronic acid is known to enhance malignancy by promoting tumor cell invasiveness and epithelial-to-mesenchymal transition [15, 16]. Following transcription, the CD44 mRNA is subject to alternative splicing, thereby giving rise to several CD44 splice variants [17]. The expression of CD44 and its splice variants, mainly variant 6 [CD44v6], has been associated with malignant transformation [18-21].

In the current study we demonstrated by genomic screening that CD44 expression is enhanced in aggressive (vasculogenic, VM^+^) tumor cells, as compared to non-aggressive (non-vasculogenic, VM^−^) tumor cells derived from both Ewing sarcoma and breast carcinoma tissues. In Ewing sarcoma patient tissues, we found a positive correlation between CD44 expression and vasculogenic blood lake presence. The observation that tumor cells adopt mechanisms of angiogenic endothelial cells to increase their chances to survive is important and suggests that targeting of CD44 is a promising anti-cancer approach.

## RESULTS

### CD44 expression is increased in aggressive vasculogenic tumor cells

To study the gene expression profile associated with vasculogenic mimicry (VM), we used the aggressive EW7 Ewing sarcoma cell line; shown to form vascular-like structures *in vitro* and *in vivo* [7] as well as the SIM.EW27 cell line, known to be less aggressive and not able to form tubes *in vitro*. To confirm proof of concept in another tumor type, the tube forming capacity of aggressive (MDA-MB-231) and non-aggressive (MCF-7) breast carcinoma cell lines was investigated (see Supplementary Figure S1). The aggressive cell line MDA-MB-231 was capable of forming tubular patterns *in vitro* on Matrigel or collagen type I matrix, whereas the MCF-7 cell line did not from any tubular structures.

**Figure 1:**
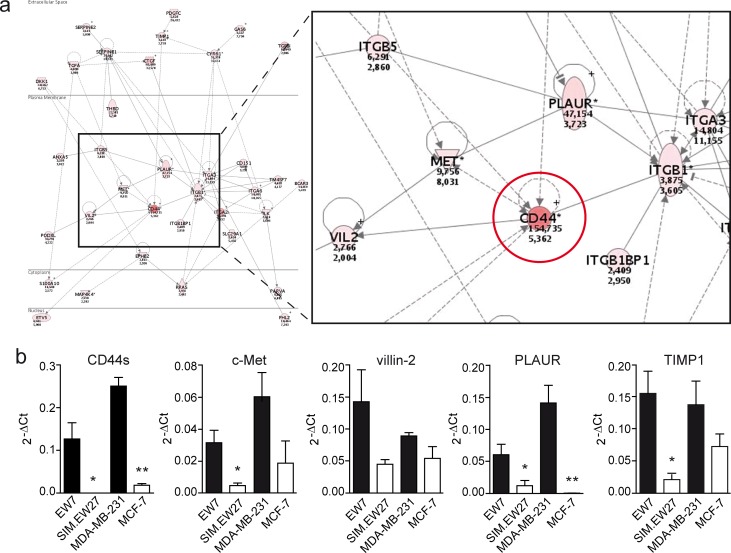
Vasculogenic tumor cells show enhanced expression of CD44/c-Met signaling components **A.** Ingenuity Pathways Analysis of microarray data showed enhanced activation of the CD44/c-Met signaling cascade. Red colors indicate enhanced expression in VM^+^ tumor cells. Encircled is CD44. **B.** Validation of the microarray data by qRT-PCR analysis of CD44s, c-Met, villin-2, PLAUR, and TIMP1 expression in the Ewing sarcoma (EW7, SIM.EW27, *n* = 4) and breast carcinoma (MDA-MB-231, MCF7, *n* = 3) cell lines. Black bars indicate VM^+^ cell lines; white bars indicate VM^−^ cell lines. Values represent the mean±SEM. *p ≤ 0.05, **p ≤ 0.01.

**Figure 2:**
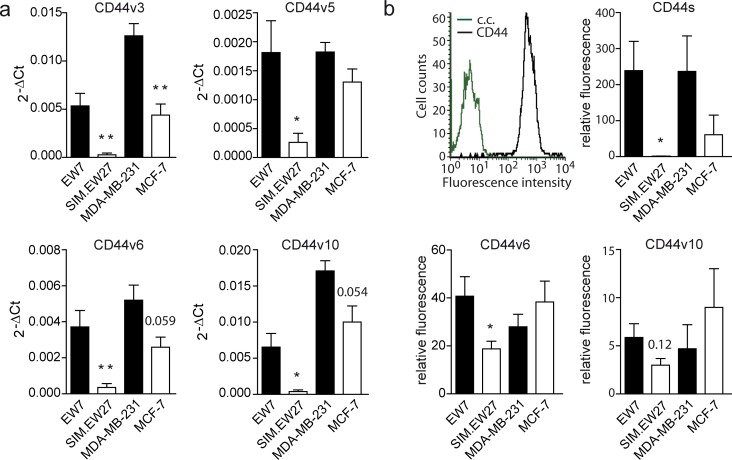
CD44 variant expression in Ewing sarcoma and breast carcinoma cell lines **A.** qRT-PCR analysis of CD44 variant expression in Ewing sarcoma (EW7, SIM.EW27, *n* = 5) and breast carcinoma (MDA-MB-231, MCF-7, *n* = 3) cell lines. **B.** Flow cytometric analysis of CD44 variant expression (*n* = 4). Upper left histogram represents the FACS plots for EW7 cells stained for CD44s (black); c.c. (green) indicates background staining of the secondary antibody. Black bars indicate VM^+^ cell lines; white bars indicate VM^−^ cell lines. Values represent the mean±SEM. *p ≤ 0.05, **p ≤ 0.01.

Next we performed microarray analysis to assess the gene signature differences between vasculogenic and non-vasculogenic tumor cells. The microarray results indicated that 84 genes were differentially and commonly overexpressed by more than 10-fold in the aggressive cell lines of both Ewing sarcoma and breast carcinoma. Array data were validated by quantitative real-time RT-PCR (qRT-PCR) for a panel of nine genes randomly selected from the list of upregulated genes (Supplementary Figure S2). Microarray data were further analyzed for gene ontology using the Database for Annotation, Visualization and Integrated Discovery (DAVID) Bioinformatics Resources 6.7 (http://david.abcc.ncifcrf.gov). Interestingly, we found a significant enrichment of genes implicated in vascular development in the aggressive cell lines versus the non- aggressive cell lines of both Ewing sarcoma and breast carcinoma tumor types. In addition, functional clustering showed enhanced presence of genes implicated in cell-matrix interactions, coagulation cascades, and cellular migration and motility. Analysis of the most upregulated genes (≥50-fold) in VM^+^ Ewing sarcoma cells revealed the presence of many vascular-related genes such as neuropilin-1, tissue factor pathway inhibitor- 2, integrins, CD44, transforming growth factor β1, and thrombospondin 1 (Table 1). Using the Ingenuity Pathways Analysis software (Ingenuity Systems, Redwood City, California, USA) we identified the CD44/c-Met signaling pathway to be crucial in the process of VM (Figure 1a). The analysis revealed that all 35 components of this signaling cascade were upregulated in the aggressive EW7 and MDA-MB-231 cell lines. Of these, CD44 was found to be the most differentially expressed gene. Five probe sets identifying (non-variant) CD44 standard (CD44s) on the array showed a robust overexpression in EW7 (on average 46.1-fold) and in MDA-MB-231 (on average 4.8-fold), as compared to their non-aggressive counterparts (Table 2). Increased expression of CD44s and four additional selected other members of the CD44/c-Met pathway were validated by qRT-PCR (Figure 1b). We also performed qRT-PCR analysis for the CD44 variants and found overexpression of variants 3, 5, 6 and 10 in vasculogenic cells (Figure 2a). Enhanced protein expression was validated in Ewing sarcoma cells for CD44s and CD44v6 using flow cytometric analysis (Figure 2b). CD44v10 showed a trend for enhanced expression in aggressive Ewing sarcoma cells, although not significantly.

**Table 1:** Functional clustering of highly upregulated genes (≥50-fold) in VM^+^ (EW7) Ewing sarcoma cells compared VM- (SIM.EW27) Ewing sarcoma cells

Functional cluster^1^	Gene ontology (GO)	Gene IDs identified in the functional cluster
Vascular development	GO:0001568 blood vessel development GO:0001944 vasculature development GO:0048514 blood vessel morphogenesis GO:0001525 angiogenesis GO:0016477 cell migration GO:0051674 localization of cell GO:0048870 cell motility	ANPEP^2^, CD44^2^, CTGF^2^, CTHRC1, HTATIP2, IL1B^2^, IL18^2^, ITGA6^2^, NRP1^2^, PLAU^2^, S100A2, QKI^2^, TGM^2^, THBS1^2^
Coagulation	GO:0042060 wound healing GO:0050817 coagulation GO:0007596 blood coagulation GO:0007599 hemostasis GO:0050878 regulation of body fluid levels	CD9, CD44^2^, DCBLD2, F2RL2, IL1B^2^, ITGA2^2^, PLAU^2^, TFPI2^2^
Cell-matrix interaction	GO:0030155 regulation of adhesion GO:0009611 response to wounding GO:0006928 cell motion GO:0050840 extracellular matrix binding GO:0045785 positive regulation of cell adhesion GO:0005178 integrin binding GO:0043236 laminin binding GO:0007155 cell adhesion GO:0022610 biological adhesion GO:0007160 cell-matrix adhesion GO:0005518 collagen binding GO:0031589 cell-substrate adhesion GO:0032403 protein complex binding GO:0009986 cell surface	ARHGDIB, CA2, CD9, CD44^2^, CTGF^2^, DCBLD2, ITGA2^2^, ITGA6^2^, IL1B^2^, IL18^2^, NRP1^2^, PERP, PTPRR, SPP1, TGFB1, TGM2, THBS1^2^

1 Functional clustering based on DAVID software analysis.

2 Genes that have been well described for their function in vascular development and/or VM.

Gene IDs: ANPEP (alanyl (membrane) aminopeptidase); ARHGDIB (Rho GDP dissociation inhibitor (GDI) β); CA2 (carbonic anhydrase II); CTGF (connective tissue growth factor); CTHRC1 (collagen triple helix repeat containing 1); DCBLD2 (discoidin, CUB and LCCL domain containing 2); F2RL2 (coagulation factor II (thrombin) receptor-like 2); HTATIP2 (HIV-1 Tat interactive protein 2); ILB1 (interleukin 1β); IL18 (interleukin 18); ITGA2 (integrin α2); ITGA6 (integrin α6); NRP1 (neuropilin 1); PLAU (plasminogen activator, urokinase); PERP (TP53 apoptosis effector); PTPRR (protein tyrosine phosphatase); S100A2 (S100 calcium binding protein A2); SPP1 (secreted phosphoprotein 1); QKI (quaking homolog, KH domain RNA binding); TGFB1 (transforming growth factor β1); TFPI2 (tissue factor pathway inhibitor 2); TGM2 (transglutaminase 2); THBS1 (thrombospondin 1).

**Table 2:** Ratio of CD44 expression between VM+ tumor cells (EW7, MDA.MB.231) and VM- tumor cells (SIM.EW27, MCF-7) based on probe set binding

Probe ID	Gene number	Gene	Gene name	Ratio EW7/SIM.EW27	Ratio MDA.MB.231/MCF-7
204489_s_at	Hs.169610.0	CD44	Homo sapiens CD44 antigen (homing function and Indian blood group system)	91,139^1^	3,966
204490_s_at	Hs.169610.0	CD44	Homo sapiens CD44 antigen (homing function and Indian blood group system)	50,108^1^	4,791
209835_x_at	Hs.169610.1	CD44	Similar to CD44 antigen (homing function and Indian blood group system)	24,049^1^	5,242
210916_s_at	Hs.306278.0	CD44	Homo sapiens CD44 isoform RC (CD44)	30,107^1^	5,197
212014_x_at	Hs.169610.3	CD44	CD44 antigen (homing function and Indian blood group system)	35,419^1^	4,542

1 present in EW7, absent in SIM.EW27.

### CD44 provides aggressive Ewing sarcoma cells with adherence capacity

**Figure 3:**
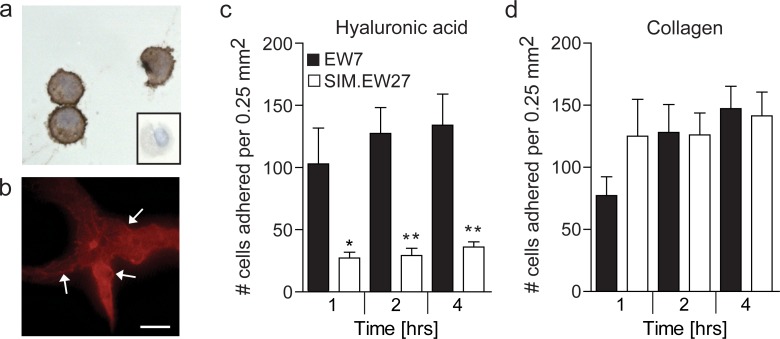
Aggressive Ewing sarcoma cells show membranous CD44 expression and gain increased adherence to the CD44 ligand hyaluronic acid **A.** Cytospin preparations of EW7 cells stained for CD44. Conjugate control is shown in the insert. **B.** CD44 staining (red) of EW7 cells that are forming vasculogenic networks on Matrigel. Arrows indicate membranous staining. Scale bar represents 100 μm. **C.** Adhesion capacity of Ewing sarcoma cell lines to hyaluronic acid or **D.** collagen after 1, 2, and 4 hours. Data in **C** and **D** are represented as mean ± SEM (*n* = 5). *p ≤ 0.05, **p ≤ 0.01.

Overexpression of CD44 in aggressive Ewing sarcoma and breast cancer cell lines, urged us to investigate functional relevance. Since we previously demonstrated that Ewing sarcoma displays the most overt presence of vasculogenic structures and Ewing sarcoma cells *in vitro* have the strongest vasculogenic capacity [7], and that the relative overexpression of CD44 in aggressive cells was highest in this tumor type, we selected Ewing sarcoma for further investigation in this study. Cytospin preparations of vasculogenic EW7 Ewing sarcoma cells showed a clear membranous staining for CD44 (Figure 3a). This was also observed for EW7 cells grown on Matrigel, on which these cells arranged in vasculogenic networks (Figure 3b). CD44 is known to contribute to tumor malignancy by modulating the adhesion of tumor cells to the extracellular matrix [22]. However, for Ewing sarcoma this functional contribution has not been examined. Therefore, we next studied the differential adherence of EW7 cells to hyaluronic acid, the natural ligand of CD44, and compared it with adherence of the non-aggressive - low CD44 expressing - SIM.EW27 Ewing sarcoma cells. We found that EW7 cells adhered more efficiently to immobilized hyaluronic acid. Moreover, attachment kinetics of EW7 showed that binding to hyaluronic acid was faster than for the non-aggressive cell line SIM.EW27 cells (Figure 3c). In contrast, the adhesion to collagen-coated plastic, which is known to depend on different adhesion molecules, did not differ between the cell lines (Figure 3d).

### CD44 is essential for vasculogenic network formation

**Figure 4:**
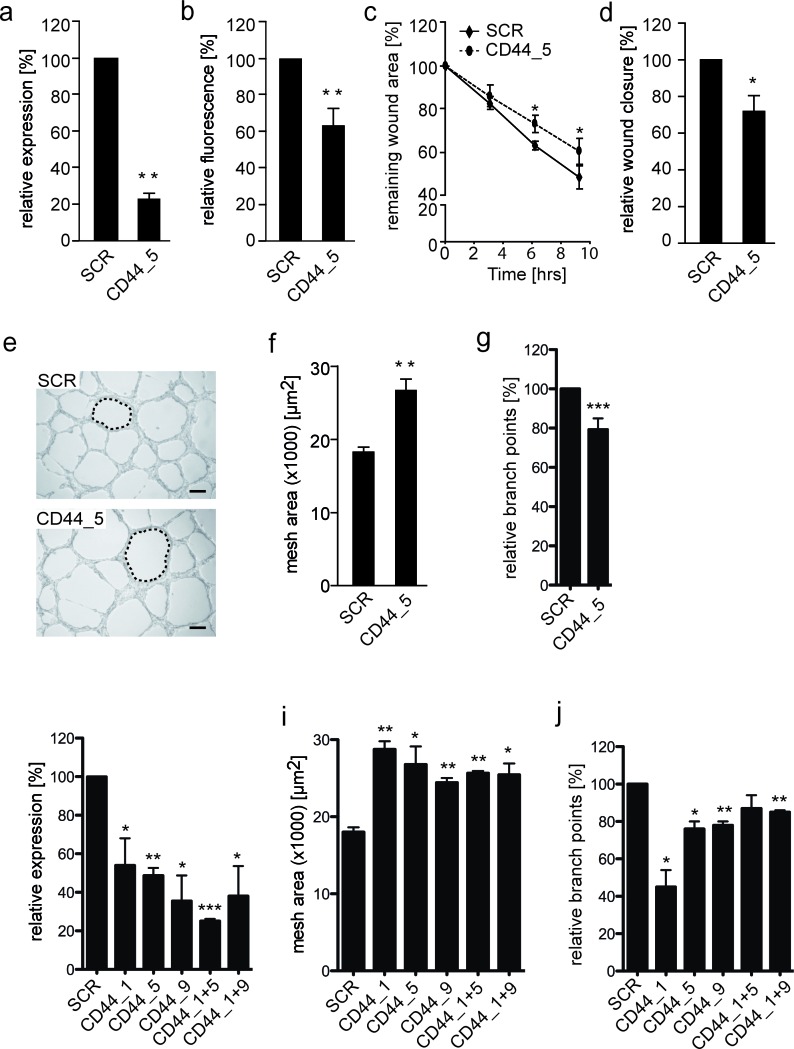
Reduced CD44 membrane expression on EW7 cells decreases tumor cell migration and vasculogenic network formation **A.** qRT-PCR and **B.** flow cytometic analysis of CD44 mRNA and protein expression, respectively, 48 hours after transfection of VM^+^ EW7 Ewing sarcoma cells with non-silencing (SCR) or CD44 targeting (CD44) siRNA. **C.** Migration analysis of transfected EW7 cells on hyaluronic acid. Relative wound closure is displayed as the percentage compared to T = 0. **D.** Relative wound closure by transfected EW7 cells as compared to SCR after 6 hours. **E.** Representative images of vasculogenic networks formed on Matrigel by SCR and CD44 targeted transfected cells. Network meshes are indicated by a dotted line. Scale bars represent 200 μm. **F.** Quantification of the average network mesh area in networks formed by SCR or CD44 transfected EW7 cells (*n* = 3). **G.** Quantification of the relative percentage of branch points. **H.** qRT-PCR analysis, 48 hours after transfection of VM^+^ EW7 Ewing sarcoma cells with non-silencing (SCR) or different CD44 targeting siRNAs (CD44_1, CD44_5, CD44_9) and combinations thereof (CD44_1+5, CD44_1+9). **I.** Quantification of average network mesh area and relative percentage of branch points **J.** of EW7 cells transfected with SCR or different CD44 siRNAs. Data are presented as mean ± SEM. *p < 0.05; **p < 0.01; ***p > 0.001.

It was subsequently aimed to determine the influence of CD44 expression on VM-characteristic network formation on a 3-dimensional matrix. Using siRNA knockdown, CD44 was suppressed by 50-75% at the RNA level, while surface protein expression was approximately 40% in the EW7 cells (Figure 4a, 4b, 4h). At this knockdown rate, diminished CD44 expression resulted in a significantly reduced migration capacity of vasculogenic Ewing sarcoma cells on hyaluronic acid (Figure 4c and 4d). Transferring the transfected cells to Matrigel revealed that patterned vasculogenic network formation by CD44 knockdown EW7 cells was suppressed as networks were less refined compared to those formed by control cells (Figure 4e). Vascular assembly was subsequently quantified based on the mean vascular mesh area. The mesh area indicates a closed region surrounded by vascular structures, i.e. an avascular zone, and is a descriptor of vascular development. The average mesh area of vascular networks formed by CD44 knockdown EW7 cells was significantly larger compared to the mesh area in vasculogenic structures formed by control cells (Figure 4f, 4i). We also counted the number of branch points to validate that sprouting indeed is reduced when CD44 is knocked down in EW7 cells (Figure 4g, 4j). These observations confirmed the functional role of CD44 in the capacity of aggressive tumor cells to form vascular structures.

### CD44 is overexpressed in clinical Ewing sarcoma tissues

**Figure 5:**
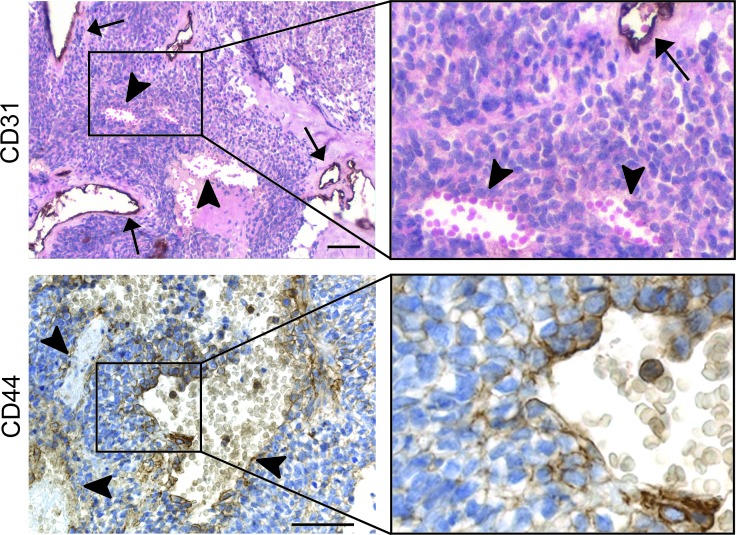
CD44 expression is related to presence of blood lakes in human Ewing sarcoma patient tissues (Upper panels) CD31 (endothelial cell) staining of Ewing sarcoma tissue section counterstained with hematoxylin (nuclei) and eosin (i.e. cytoplasm, erythrocytes). The magnification of the insert is displayed on the upper right. Arrows indicate regular blood vessels; arrowheads show VM-characteristic blood lakes. (Lower panels) CD44 staining on Ewing sarcoma cells enclosing blood lakes. The right images are an enlargement of the respective inserts of the left images. Scale bars indicate 50 μm.

To verify our *in vitro* findings, we stained a set of human Ewing sarcoma tissues for CD44. Interestingly, we found a significant correlation between the presence of blood lakes (Figure 5, upper panels), a characteristic of aggressiveness and an appearance of VM [7], and expression of CD44. Note here that blood lakes are negative for the vascular marker CD31 as indicated by the arrowheads (Figure 5, upper panels). Collectively, CD44 was found to be expressed in 14 out of 15 Ewing sarcoma tissues scored positive for the presence of blood lakes (93% of cases, Table 3, *p* < 0.002). As we described previously the presence of blood lakes is negatively correlated to survival [7]. CD44 expression might therefore also be considered a predictor of short survival. Although CD44 is a marker of leukocytes, histological analysis at higher magnification demonstrated that CD44 was expressed by those tumor cells directly lining the blood lakes (Figure 5, lower panels), as was similarly described for vascular endothelial (VE)-cadherin, a well-defined marker of VM [7].

**Table 3:** CD44 expression in human Ewing sarcoma tissues

Sample ID	Blood lakes	CD44^1^
A15899	+	++
690047	+	−
1017783	+	++
1115689	+	+
1111044	+	+
659137	+	+
1070335	+	+
668292	++	++
697664	+	+
04824	+	+
09978	+	+
4755	+	+
8677-3	+	+
10265-1	+	+
6511-2	+	+
654766	−	−
103970715	−	−
1115539	−	−
696359	−	+
NB2316	−	−
105794	−	−
7167-1	−	+
6476-2	−	−

1 CD44 scoring is based on tumor cell staining; − absent; + present; ++ highly present.

## DISCUSSION

In the present report, we showed that CD44 expression is increased in aggressive, vasculogenic network forming tumor cells. Functional activity of CD44 in these cells, i.e. adhesion to hyaluronic acid, suggests that this molecule is involved in enhanced aggressiveness through vasculogenic mimicry (VM). Indeed, knockdown of CD44 resulted in reduced tumor cell migration and suppressed network formation *in vitro*. In addition, CD44 expression was found to heavily correlate with the presence of blood lakes in tumor tissues of patients with Ewing sarcoma. In these clinical samples CD44 expression was associated with tumor cells directly lining the vasculogenic structures.

Transdifferentiation of plastic tumor cells into a vasculogenic phenotype allows their contribution to blood circulation [1, 7] and provides a tumor with a higher level of autonomy, further escape from host regulation, and therefore higher aggressiveness. This phenomenon is strongly associated with enhanced malignancy and shorter patient survival, independent of tumor type [2-6].

CD44 was originally identified as an adhesion molecule or homing receptor involved in leukocyte recirculation [23]. It is known in cancer cell biology that tumor cells are able to copy this recirculation mechanism in order to gain cellular motility and the capacity to metastasize [17]. Tumor cells are not the only cells that use this mechanism for enhanced motility. On endothelial cells, CD44 is known as a marker of tumor angiogenesis, and its adhesion function facilitates endothelial cell migration and sprouting [14]. It is therefore plausible that tumor cells that want to obtain endothelial cell-like characteristics induce the expression of CD44 to enable them to form vasculogenic structures. In agreement with this, we found that reduced expression of CD44 on vasculogenic tumor cells interfered with their vasculogenic network assembly. Although CD44 knockdown tumor cells kept their ability to form vascular structures, the capacity to do so was significantly reduced. This demonstrated the functional contribution of CD44 during vascular network formation. As we achieved a maximal protein knockdown efficacy of 40%, more effective targeting of this protein may severely hamper VM.

The role of CD44 during endothelial-related processes has been described in relation to c-Met signaling. CD44v6 was found to control endothelial cell sprouting and migration induced by the activation of c-Met [24, 25], while CD44v10 was reported as a regulator of c-Met-mediated vascular barrier integrity. Interestingly, our microarray data suggested a critical involvement of the whole CD44/c-Met signaling cascade during VM. The c-Met oncogene encodes the tyrosine kinase receptor for hepatocyte growth factor (HGF) and in tumor cells the constitutive activation of c-Met is associated with enhanced growth, invasion, and survival [26]. Several reports have described the capacity of CD44 variants to promote or increase c-Met activation [27, 28]. Although the interaction of CD44 (variants) with c-Met has not been examined in this study, the above indicates that both molecules are important for proper endothelial function. Therefore, tumor cells that have adopted an endothelial phenotype may benefit from the expression of these proteins.

Interestingly, although vasculogenic tumor cells gain endothelial features, they do not gain sensitivity to angiogenesis inhibitors, as others and we have clearly demonstrated previously [9, 29]. Although the occurrence/presence of VM in tumors has been highly debated (reviewed in [30, 31]) this finding may imply that VM is a resistance mechanism against angiostatic compounds [32]. It has even be hypothesized that anti-angiogenic therapy might induce the formation of VM [33]. Thus our results profoundly indicate that a targeting strategy against VM is most urgently needed. The results of the current study suggest that CD44 would be an ideal target for therapy, as it would target multiple aspects of tumor biology. Firstly, CD44 inhibition would target the most aggressive, most dedifferentiated, vasculogenic tumor cells. Secondly, targeting CD44 would attenuate tumor angiogenesis, thus decreasing blood and nutrient supply to the tumor cells. The combination of angiogenesis inhibition and CD44 targeting would prevent resistance, by means of VM, against the angiostatic compound. Thirdly, the advantage of a CD44-based anti-cancer strategy would be the targeting of the tumor stem cell compartment. Besides VM, other tumor features like epithelial-to-mesenchymal transition and stemness depend heavily on the capacity of tumor cells to gain a trans- or dedifferentiated phenotype. The presence of these features within a tumor is related to increased tumor malignancy, and this association is well documented for VM [4]. Importantly, CD44, which we found to be overexpressed in vasculogenic tumor cells, has been well described in relation to both epithelial-to-mesenchymal transition and tumor-initiating stem cells. In transformed human mammary epithelial cells, induction of epithelial-to-mesenchymal transition generated cells with a CD44^high^/CD24^low^ antigenic phenotype. This subset of cells is greatly enriched in the tumor-initiating cell (cancer-stem cell) population [34]. In addition, CD44 is a well-described tumor stemness marker. In breast and prostate carcinoma, CD44 is commonly used as a surface marker to identify cancer stem-like or progenitor cells, i.e. tumor cells with self-renewal potential [35-37]. Recently, it was demonstrated that glioblastoma stem-like cells are able to differentiate towards an endothelial lineage [38]. Interestingly in addition to glioblastoma, a similar endothelial potential may be shared by CD44-expressing cells isolated from ovarian cancer [39]. The above demonstrates an important function for CD44 in retaining a trans- or dedifferentiated phenotype by aggressive tumor cells. Interestingly, the ability of plastic tumor cells to engage in VM is associated with their ability to express key pluripotent stem cell markers, and activate embryonic signaling pathways [9, 10, 40]. This supports our data demonstrating the expression of CD44 on vasculogenic tumor cells, as these cells have aborted their original cell-specific lineage. Moreover, it suggests a function for CD44 as a gatekeeper of this newly adopted phenotype and a marker of increased tumor malignancy through tumor cell plasticity.

Although various studies have correlated the expression of CD44 to increased tumor malignancy, some studies did not find a positive association between CD44 expression or activation and tumor progression [41-43]. Another argument against a CD44-based targeting strategy is its expression in non-neoplastic cells of various tissue origin [44], such as leukocytes. Therefore, serious side effects may be expected. However, full ablation of immune cells is not expected, as pluripotent haematopoietic stem cells do not express CD44. Recently a phase I clinical study with an anti-CD44 antibody in patients with malignant solid tumors (https://clinicaltrials.gov; NCT01358903) was completed. The results of this study were not available yet at this writing. The study was designed to target tumor cells in general. However, it should be realized that the antibody might exclusively target vasculogenic tumor cells. As VM occurs in many different tumor types and is co-existent with angiogenesis [8, 45] monotherapy targeting VM might not be sufficient to inhibit tumor growth. Instead combination therapy of angiogenesis inhibitors and VM targeting drugs should be tried. Furthermore, vasculogenic mimicry is a predictor of poor clinical outcome [45, 46] and it has become increasingly clear that novel treatment strategies, which target tumor cell plasticity, are needed [30, 45].

In conclusion, our results identified CD44 as a novel marker of vasculogenic tumor cells and show an important function for CD44 in the formation of vascular-like tumor cell networks. Although its expression has previously been found to correlate with increased tumor malignancy and tumor stemness, CD44 has not been associated with VM before. Our findings offer new opportunities for future treatment strategies, as targeting CD44 may not only affect VM, but may also inhibit tumor angiogenesis and target the cancer stem cell population.

## MATERIALS AND METHODS

### Cell lines and 3-dimensional cell culture

Ewing sarcoma cell lines EW7 and SIM.EW27 were previously characterized by Dr. O. Delattre (Pathologie Moléculaire des Cancers, Institut Curie, Paris Cedex). MDA-MB-231 and MCF-7 breast carcinoma cell lines were obtained from the American Type Culture Collection (ATCC). Cells were maintained in RPMI-1640, supplemented with 10% fetal calf serum and 2 mM L-glutamine. Cells were grown on standard culture dishes except for the SIM.EW27, which were grown on gelatin-coated culture dishes.

Vasculogenic tube formation was tested using a 3-dimensional gel system. Wells from a 96-well plate were coated with 40 μl Matrigel (BD Biosciences, Woerden, the Netherlands) at 37 °C after which cells were plated at 20 000 cells per well and incubated for 16 hours to study vasculogenic network formation. At this time point photographs were taken using a microscope (DMI3000B, Leica Microsystems B.V., Rijswijk, the Netherlands) equipped with an integrated camera. The images were quantified for the mean area value of randomly selected vascular network meshes using Image J 1.41. Network meshes were defined as a closed area surrounded by branching structures, i.e. avascular zones. For 3-dimensional collagen cultures, wells from a 24-well plate were coated with 25 μl collagen type I (Vitrogen, Nutacon B.V., Leimuiden, the Netherlands). The collagen was dehydrated using 70% ethanol (5 minute incubation), which was followed by 3 washings with PBS. Subsequently 40 000 cells were plated in each well. The cells were allowed to grow confluent and the medium was replaced every day. Tubular patterns (when formed) were visible after 6-7 days at which time point pictures were taken.

### Antibodies and materials

In this study, the following antibodies were used: anti-CD44 standard clone Hermes-3 (kindly provided by S. Jalkanen, Turku, Finland), CD44v3 clone VFF-327v3 (Bender Medsystems, Vienna, Austria), CD44v5 clone VFF-8, CD44v6 VFF-7 and VFF-18 (Bender Medsystems), CD44v10 clone VFF-14, anti-CD31 (DakoCytomation, Glostrup, Denmark) and anti-CD34 (Novocastra, Valkenswaard, the Netherlands). The secondary antibodies were biotinylated anti-mouse polyclonal antibodies as described in the methods for immunohistochemistry and flow cytometry.

### Microarray analysis

Total RNA was isolated from the cell lines EW7, SIM.EW27, MDA-MB-231, and MCF-7 using the RNeasy RNA isolation kit (Qiagen, Venlo, the Netherlands). Possible genomic DNA contaminations were eliminated by on column RNase-free DNase treatment (Qiagen). RNA samples quantity and purity were determined using the Nanodrop ND-1000 spectrophotometer (Nanodrop Technologies, Wilmington, USA) and RNA integrity was determined using the Bioanalyzer2100 (Agilent Technologies, Palo Alto, USA). RNA was amplified using the two-cycle cDNA synthesis kit (Affymetrix, Santa Clara, USA) in combination with the MEGAscript T7 *in vitro* transcription system (Ambion, Foster City, US). Biotin labeled target complementary RNA was fractionated and hybridized to Human Genome U133A Plus 2.0 Arrays (Affymetrix). Each of these arrays contained >54 000 oligonucleotide probe sets corresponding to 38 500 characterized genes. Image data were analyzed using Affymetrix GeneChip^®^ Operating Software (GCOS) version 1.4. For each transcript represented on the array, the expression algorithm computed the detection call (present, marginal, or absent), the detection p-value, and the average signal intensity value for each probe set. Transcripts differentially expressed (threshold of 2-fold) between aggressive (VM^+^) and non-aggressive (VM^−^) cell lines were used for further analysis. Gene identifiers and corresponding expression values of those transcripts were uploaded into Ingenuity Pathways Analysis (Ingenuity^®^Systems, www.ingenuity.com). These data were overlaid onto a global molecular network developed from information contained in Ingenuity's Knowledge Base. Networks were then algorithmically generated based on their connectivity.

### Quantitative real-time RT-PCR

Total RNA was isolated from tissue sections using RNeasy (Qiagen), followed by RNase-free DNase treatment (Qiagen). RNA concentration was measured using the Nanodrop ND-1000 spectrophotometer (Nanodrop Technologies). cDNA was reverse transcribed using MMLV-RT (Bio-Rad, Veenendaal, the Netherlands) according to manufacturers' instructions. Quantitative real-time RT-PCR (qRT-PCR) was performed with primer sequences listed in Supplemental Table S1 with cyclophilin A and β-actin primers as reference genes (Eurogentec, Liege, Belgium). qRT-PCR was performed using an iCycler MyIQ (Bio-Rad) in 25 μl volume containing 20 ng cDNA, 1×SYBR^®^ Green PCR master mix (Eurogentec) spiked with 20 nM fluorescein (Bio-Rad), and 400 nM of each primer. Data was analyzed with the Sequence Detection System software (Applied Biosystems, Foster City, US).

### Flow cytometry

After trypsinization, cells were fixed in 1% paraformaldehyde for 30 minutes at room temperature and incubated with primary antibodies directed to CD44s or CD44 variants appropriately diluted in PBS/0.1% bovine serum albumin. Following one-hour incubation on ice the cells were washed and subsequently incubated with biotinylated rabbit-anti-mouse IgG (DakoCytomation) followed by streptavidin-PE or streptavidin-FITC (DakoCytomation). Cells were analyzed using the FACSCalibur Flow Cytometer (BD Biosciences). Data were analyzed using CellQuest^TM^ software.

### Adhesion

Cells were harvested by trypsinization and counted. 10 000 cells were applied to a coated (5 mg/ml hyaluronic acid versus 3mg/ml collagen) 96-well plate and allowed to adhere for 1, 2, or 4 hours. The non-adhered cells were washed away and the adhered cells were counted per 0.25 mm^2^ field.

### Migration assay

Cells were plated in 5 mg/ml hyaluronic acid-coated 96-well plates at 40 000 cells per well and allowed to grow to confluence overnight. Wells were uniformly scratched using a guided 96-well pin tool (Peira, Turnhout, Belgium) to create wounds. Subsequently, wells were washed with PBS and fresh medium was added. Images were automatically captured with a Leica DMI3000 microscope (Leica, Rijswijk, the Netherlands) using Universal Grab 6.3 software (DCILabs, Keerbergen, Belgium). Scratch sizes were determined using Scratch Assay 6.2 (DCILabs), and wound closure was analyzed.

### Transfection

EW7 cells were transiently transfected with siRNA targeting CD44, or non-silencing (scrambled, SCR) siRNA (Qiagen). The different CD44 targeting siRNAs tested were: CD44_1 (SI 00012775), CD44_5 (SI 00299705) and CD44_9 (SI 03062661). In wells from a 24-well plate, 20 nM siRNA was mixed with 1 μl of HiPerFect Transfection Reagent (Qiagen). The mixture was allowed to complex for 15 minutes at room temperature. Afterwards cells were plated at 35 000 cells per well and grown for 48 hours. Subsequently, cells were either fixed with 1% paraformaldehyde for flow cytometry analysis or used for Matrigel assay (see section “Cell lines and 3-dimensional cell culture”) or migration analysis (see section “Migration analysis”).

### Immunohistochemistry

A collection of 23 patient samples was examined to study blood lakes and CD44 expression. These patient tissues were previously described in the study of van der Schaft *et al* [7], and selected based on tissue availability. Tumor tissues were obtained from the University of Leuven and the Leiden University Medical Center. Patients presented with Ewing sarcoma between 1987 and 2004. Most patients were included in the European Intergroup Cooperative Ewing's Sarcoma Study and Euro-EWING studies [47]. Additionally sections from patients from the University of Gent were included. All patient materials were handled in a coded fashion according to the protocols as detailed by the Dutch association of Medical Scientific Associations.

For immunohistochemistry, paraffin sections were dried for 48 hours at 37 °C prior to staining. Tissues sections were deparaffinized and rehydrated. Endogenous peroxidase activity was blocked by immersing the slides in 0.3% H_2_O_2_ in methanol for 20 minutes. Heat-induced epitope retrieval was performed by autoclave treatment in citrate buffer. Sections were washed with PBS and aspecific binding sites were blocked with 5% bovine serum albumin in PBS. Primary antibody binding was detected using biotinylated anti-mouse Ig antibodies (DakoCytomation) and visualized by streptavidin ABComplex/HRP (DakoCytomation) and 3,3′-diaminobenzidine (DAB) as substrate (Sigma-Aldrich Chemie B.V., Zwijndrecht, the Netherlands). Afterwards, tissues were counterstained with hematoxylin (and eosin) and mounted with non-aqueous DePeX mounting medium (Serva Electrophoresis, Heidelberg, Germany). Histological sections were viewed at room temperature using an Olympus BX50 microscope equipped with a camera (Leica, DC300). Images were processed using Photoshop (Adobe) and figures were made in Illustrator (Adobe).

### Scoring of Ewing sarcoma tissues

The VM structures were detected in the hematoxlin/eosin, CD31 stained tumor tissues. VM structures (blood lakes) were defined as the irregularly lined areas containing erythrocytes, but lacking the presence of CD31-positive endothelial cells. The number of blood lakes per tumor area was scored. Scoring for CD44 in the blood lakes was based on the intensity of the tumor cell staining. – absent; + present; ++ highly present

### Statistical analysis

All data are expressed as mean values ± standard error of the mean (SEM). Statistical analyses were performed using a Student's *t* test (GraphPad Prism 5, GraphPad Software Inc., San Diego, CA). P-values ≤ 0.05 were considered statistically significant.

## SUPPLEMENTARY FIGURES AND TABLE


