# Supplementary material for: CD44 enhances tumor aggressiveness by promoting tumor cell plasticity
Source: Oncotarget. 2015 Apr 15;6(23):19634–46. doi: 10.18632/oncotarget.3839 (PMC4637310; doi:10.18632/oncotarget.3839)
Supplement: Supplementary file 1 [file oncotarget-06-19634-s001.pdf]

CD44 enhances tumor aggressiveness by promoting tumor cell plasticity

Supplementary Material

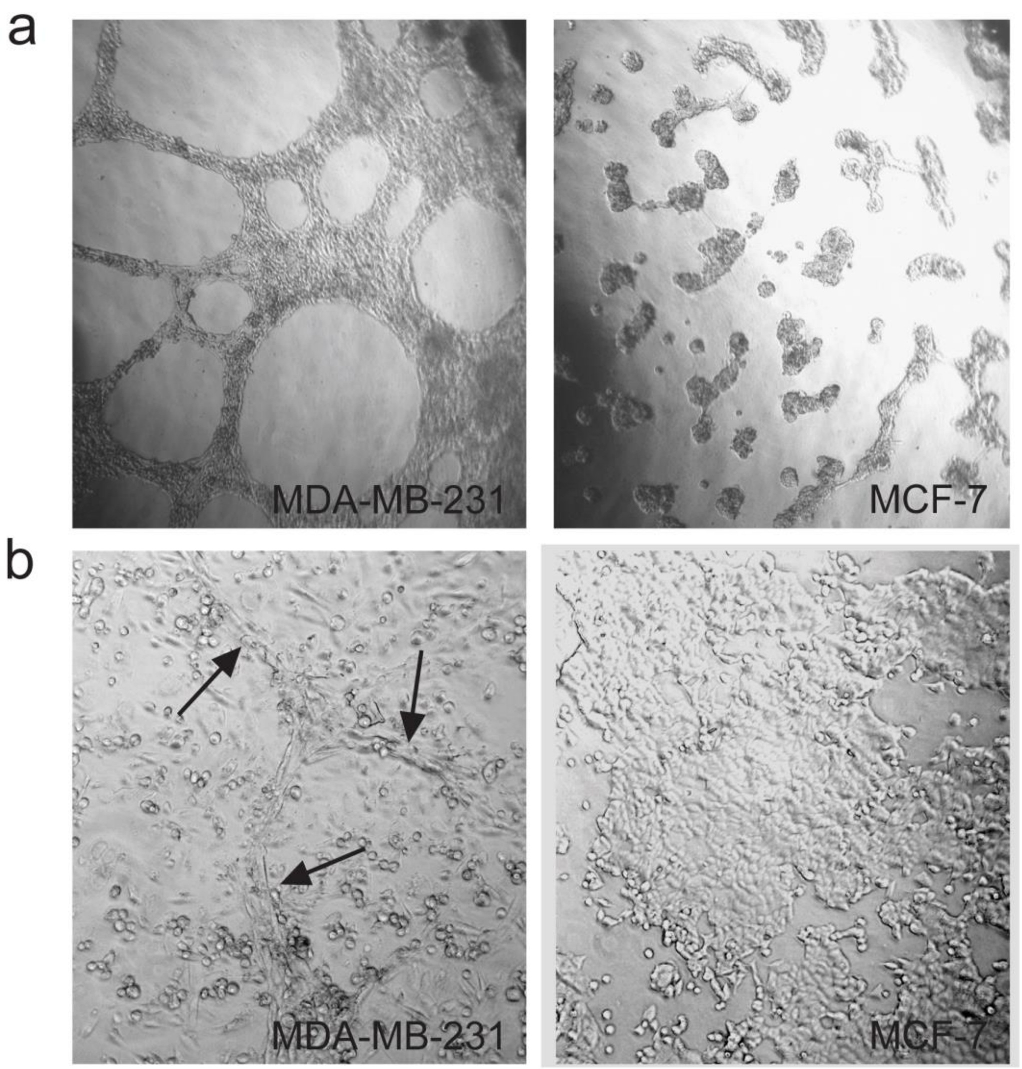

Supplementary Figure S1: MDA-MB-231 breast carcinoma cells are capable of VM. (**a**) MDA-MB-231 cells, in contrast to MCF-7 cells, form tubular patterns when cultured *in vitro* on Matrigel or (**b**) on collagen type I.

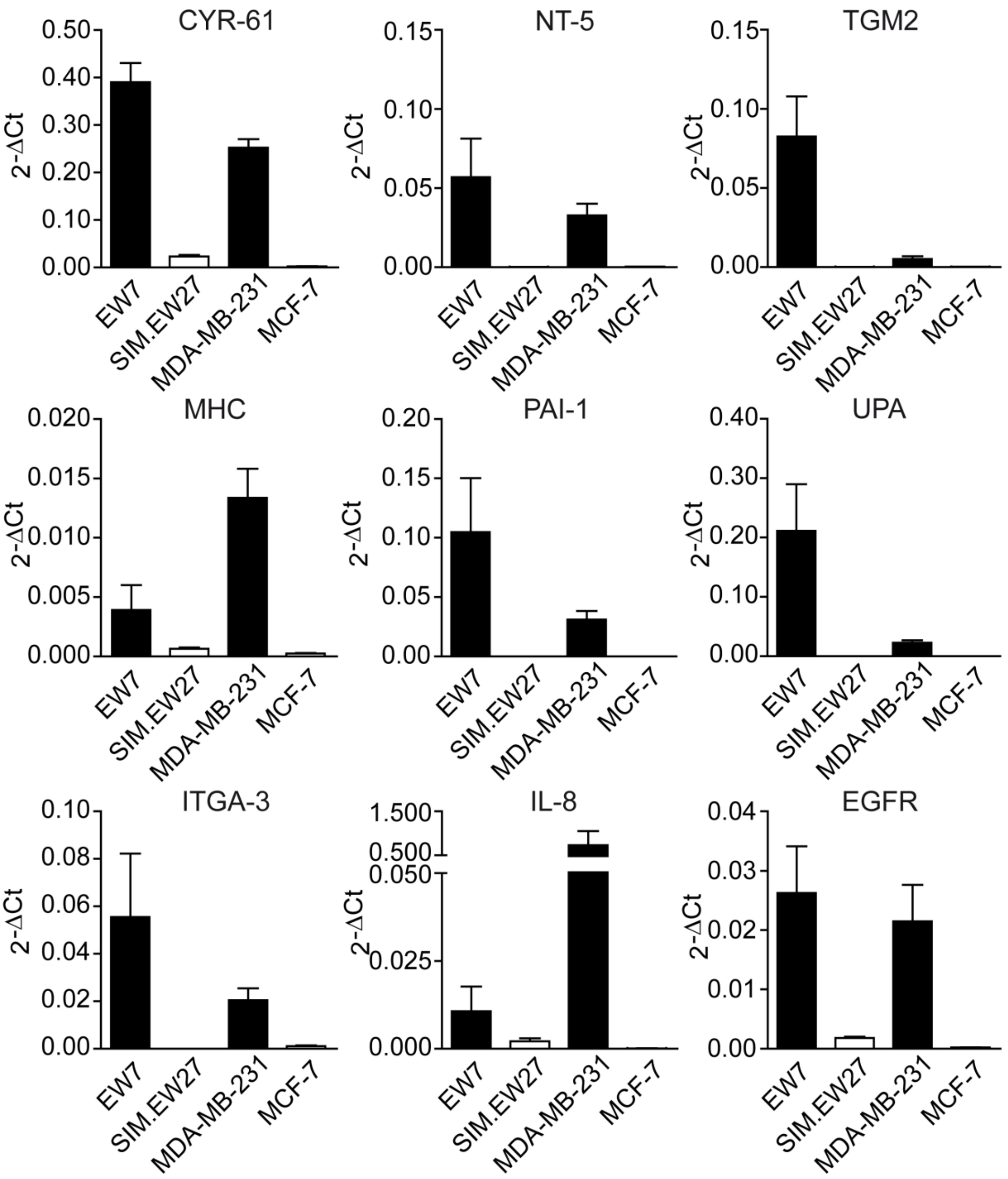

Supplementary Figure S2: Validation of microarray. Expression analysis of a selection of genes identified by microarray to be overexpressed by more than 10-fold in both VM$^+$ Ewing sarcoma (EW7) and breast carcinoma (MDA-MB-231) cell lines as compared to the VM$^-$ Ewing sarcoma (SIM.EW27) and breast carcinoma (MCF-7).

Supplementary Table S1: Primer sequences.

Gene	Forward 5’→3’	Reverse 5’→3’
β-actin	GCTGTGCTACGTCGCCCTG	GGAGGAGCTGGAAGCAGCC
Cyclophilin A	CTCGAATAAGTTTGACTTGTGTTT	CTAGGCATGGGAGGGAACA
CD44s	GCAGTCAACAGTCGAAGAAGG	CCGTTGAGTCCACTTGGC
CD44v3	AAATACCATCTCAGCAGGCT	TGGTGCTGGAGATAAAATCTT
CD44v5	ATGTAGACAGAAATGGCACCA	TTGTAGAATGTGGGGTCTCTT
CD44v6	AGTACAACGGAAGAAACAGCTA	TGTCCCTGTTGTCGAATGG
CD44v10	ATAGGAATGATGTCACAGGTGG	TCCTTCGTGTGTGGGTAATG
PLAUR	CAGTGCCTGGATGTGA	AGGAAGTGGAAGGTGTCGTT
c-Met	GATGGCAAGAAAATTCACTGT	CTTCACTTCGCAGGCAGAT
Villin-2	ACACTGCCAAGATTGCCC	GTCATCACCAGGTGCAGCT
TIMP1	AGTGTCTGCGGATACTTCCA	CTAAGCTCAGCCTGTTCCAG
